# Characterization of Eight Novel Spiroleptosphols from *Fusarium avenaceum*

**DOI:** 10.3390/molecules24193498

**Published:** 2019-09-26

**Authors:** Klaus Ringsborg Westphal, Manuela Ilse Helga Werner, Katrine Amalie Hamborg Nielsen, Jens Laurids Sørensen, Valery Andrushchenko, Jacob Winde, Morten Hertz, Mikkel Astrup Jensen, Mathilde Lauge Mortensen, Petr Bouř, Teis Esben Sondergaard, Reinhard Wimmer

**Affiliations:** 1 Department of Chemistry and Bioscience-Section for Biotechnology, Aalborg University, Frederik Bajers Vej 7H, 9220 Aalborg Ø, Denmark; kw@bio.aau.dk (K.R.W.); manuelasandmann@gmx.com (M.I.H.W.); kah@bio.aau.dk (K.A.H.N.); jacobwinde@gmail.com (J.W.); hertzmorten@gmail.com (M.H.); mijens11@gmail.com (M.A.J.); mathilde.91@hotmail.com (M.L.M.); tes@bio.aau.dk (T.E.S.); 2 Department of Chemistry and Bioscience, Aalborg University, Niels Bohrs Vej 8, 6700 Esbjerg, Denmark; jls@bio.aau.dk; 3 Institute of Organic Chemistry and Biochemistry, Czech Academy of Sciences, Flemingovo náměstí 542/2, 166 10 Prague 6, Czech Republic; petr.bour@uochb.cas.cz

**Keywords:** spiroleptosphol, secondary metabolites, polyketides, polyketide synthases, PKS, *Fusarium avenaceum*

## Abstract

Chemical analyses of *Fusarium avenaceum* grown on banana medium resulted in eight novel spiroleptosphols, T1, T2 and U–Z (**1**–**8**). The structures were elucidated by a combination of high-resolution mass spectrometric data and 1- and 2-D NMR experiments. The relative stereochemistry was assigned by ^1^H coupling and NOESY/ROESY experiments. Absolute stereochemistry established for **7** by vibrational circular dichroism was found analogous to that of the putative polyketide spiroleptosphol from *Leptosphaeria doliolum*.

## 1. Introduction

Filamentous fungi of the genus *Fusarium* are notorious plant pathogens, causing serious losses of grain every year world-wide. In addition, the fungi produce several harmful compounds during plant infection, which can cause illnesses in both humans and animals. Some of these natural products belong to the group of polyketides (PKs), which are biosynthesized by enzymatic machinery based on the polyketide synthases (PKSs). More than 65 different genes encoding PKSs have so far been identified across the *Fusarium* genus, of which *F. avenaceum* is one of the strains with most PKS genes but only a few products are known [1,2]. PKs are a structurally diverse group of compounds named after their biosynthetic origin, where they are assembled from small malonyl and acetyl subunits. Examples of beneficial and harmful PKs from *Fusarium* are fusarielins, aurofusarin and gibepyrons, which have been shown to have an estrogenic effect on cancer cells, inhibiting probiotic bacteria and nematocidal activity, respectively [3,4,5,6,7]. The secondary metabolome from fungi seldom includes all PKs that potentially could be produced, hence different approaches are needed for activation of responsible gene clusters. One way to trigger gene activation is utilizing different media compositions termed the OSMAC (one strain many compounds) approach [8,9]. A considerable difference between secondary metabolite profiles in *fusaria* has been demonstrated previously by altering the yeast extract from different suppliers in the growth media or by growth on unusual media [10,11].

**Figure 1:**
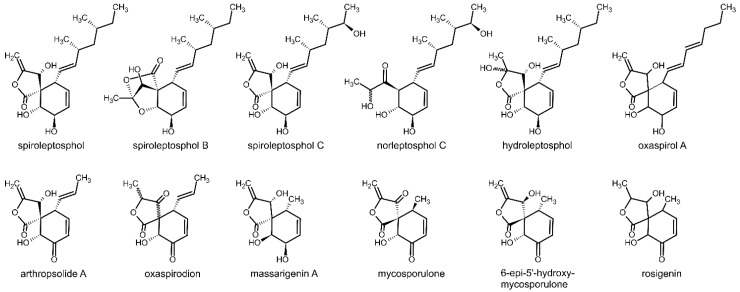
Spiroleptosphols and analogous compounds known from literature.

To document the secondary metabolite potential of *Fusarium* when grown on different media, we isolated eight related PKs not reported before (**1**–**8**). For this purpose, *F. avenaceum* was grown on banana agar medium. Below, we describe in detail the structure elucidation of **1**–**8** using HRMS and NMR. The relative and absolute stereochemistry was established by NMR and vibrational circular dichroism (VCD) spectroscopies. These eight compounds have similar structural features with spiroleptosphol, a cytotoxic γ-methylindene-spiro butanolide isolated from the ascomycetous fungus *Leptosphaeria doliolum* and analogous thereof (Figure 1) [12,13,14,15,16,17,18,19,20,21,22,23]. Therefore, they were named spiroleptosphol U–Z, with spiroleptosphol T existing in two diastereomers called T1 and T2.

## 2. Results and Discussion

**Figure 2:**
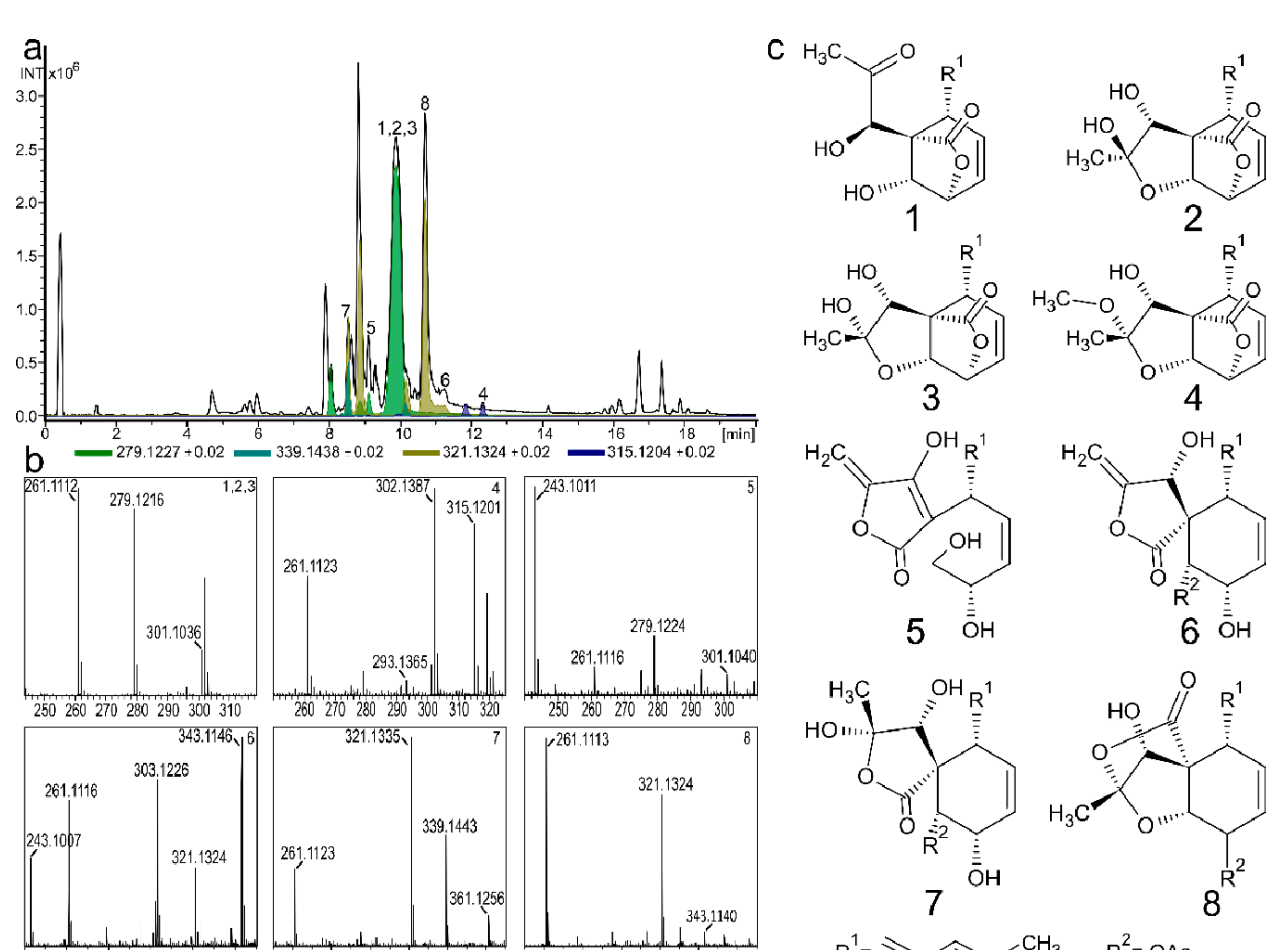
HPLC-HRMS of a pre-fractionated metabolite extract from *F. avenaceum* grown on banana media showing **1**–**8**. (**a**) Base peak chromatogram (black) and colored extracted ion chromatograms of **1**–**8**. **1**–**3** and **5** [M + H]^+^ EIC = 279.1227 Da. **4** [M + Na]^+^ EIC = 315.1204 Da. **6** and **8** [M + H]^+^ EIC = 321.1324 Da. (**7**) [M + H]^+^ EIC = 339.1438 Da.; (**b**) six mass spectra showing ion adducts from compounds **1**–**8**.; (**c**) Polyketides **1**–**8**.

In an effort to identify the chemical nature of *Fusarium* secondary metabolism, several isolates were grown on a variety of different media. The resulting metabolite extracts were profiled using HPLC-DAD-HRMS. Several unusual metabolites were observed (*m*/*z* between 250–350 Da) when *F. avenaceum* strain 05001 was cultivated on solid banana medium (Figure 2a,b). Augmented cultivation of *F. avenaceum* and metabolite extraction followed by compound isolation by semi-preparative RP chromatography resulted in PKs **1**–**8** (Figure 2c). The structures of **1**–**8** were all elucidated by 1D- and 2D NMR spectroscopy and the absolute configuration was assigned by VCD for **7**.

### 2.1. Spiroleptosphol U, T1 and T2 (**1**, **2** and **3**)

**Figure 3:**
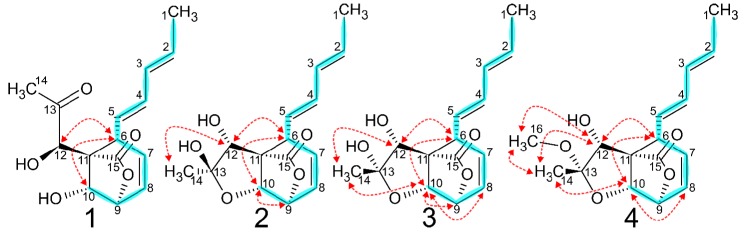
Homonuclear 2D NMR correlations of **1**–**4** showing TOCSY/COSY (blue) and NOESY/ROESY (red) correlations. TOCSY, COSY and NOESY was applied for **1**–**3** while COSY and ROESY was applied for **4**.

Compounds **1**–**3** eluted as a single broad peak when analyzed by HPLC-HRMS. This would imply an equilibrium with relatively fast interchange between compounds. The HR-ESI(+)MS returned a [M + H]^+^ at 279.1216 Da and a [M − H_2_O + H]^+^ at 261.1112 Da confirmed by a sodium adduct [M + Na]^+^ at 301.1036 Da. This, together with the isotope distribution, was consistent with a chemical formula of C_15_H_18_O_5_. 1D and 2D NMR experiments revealed three compounds with similar characteristics of ^1^H and ^13^C chemical shifts, ^1^H–^1^H couplings, COSY and ^1^H–^13^C HMBC correlation (Table 1). For all three compounds, TOCSY/COSY correlations were observed for a series of ten carbon atoms initiating at a methyl group (H^1^) and proceeding through two *trans-*alkenes (H^2−5^), a methine (H^6^), a *cis*-alkene (H^7−8^) and ending with two methines (H^9−10^) both with ^13^C chemical shifts resonating between 75 and 84 ppm indicating bonding to oxygen. HMBC correlations from H^6^ and H^10^ to a quaternary carbon (C^11^) formed a six-carbon ring from C^6^ to C^11^ (Figure 3). Additionally, a resonance between 176 and 178 ppm (C^15^) displayed HMBC correlations to C^9^ and C^11^ and can only be explained by forming a γ-lactone ring consisting of C^9^–C^11^, C^15^ and O^9^. Finally, a methine (C^12^) observed as a singlet showed HMBC correlations to C^11^. ^1^H and ^13^C chemical shifts of C^12^ suggested bonding to an oxygen. For Compound **1**, a resonance for C^13^ (δ_C_ 212.6) was attributed to a ketone which was not observed for **2** and **3**.

HMBC correlations placed C^13^ between C^11^ and a methyl group (C^14^). Two hydrogens could not be observed in the ^1^H spectrum, thus these could be assigned to two alcohols (C^10^ and C^12^), fulfilling the determined molecular mass, thereby giving the structure of **1**. The relative stereo configuration of **1** was assigned by NOESY correlations between H^6^–H^10^ and H^6^–H^12^. Compounds **2** and **3** both showed a quaternary carbon (C^13^) resonating at 109.6 and 104.7 ppm, respectively, in place of the ketone in **1**. This relatively high chemical shift was explained by bonds to two oxygens, one as an alcohol and the other as an ether to C^10^ giving a third ring and resulting in the diastereomeric structures of **2** and **3**. The differences in ^13^C chemical shifts between **2** and **3** was increasing around C^13^ indicating a steric inversion at this position. This was supported by the presence of a NOESY correlation between H^10^ and H^14^ in **3** which was not present in **2**. Otherwise, the relative stereo configuration was identical to that of **1**. In addition, the NOESY spectrum displayed cross peaks with opposite signs, indicating their origin from chemical exchange. Those cross peaks were observed between H^9^ of **1** and H^9^ of both **2** and **3,** but not between H^9^ of **2** and **3**. The same pattern was seen for H^10^ and H^12^. Also, H^6^ displays an exchange peak between **1** and **2**/**3**, but due to almost identical chemical shifts, the presence or absence of an exchange peak between H^6^ of **2** and **3**, respectively, cannot be established. This indicates that **1** is in equilibrium with both **2** and **3**.

### 2.2. Spiroleptosphol W (**4**)

HR-ESI(+)MS of **4** returned a [M + H]^+^ at 293.1365 Da as well as ion adducts of [M − CH_4_O + H]^+^ at 261.1123 Da, [M + Na]^+^ at 315.1201 Da and a [M − H_2_O + C_2_H_3_N + H]^+^ at 302.1387 Da. Including the isotopic distribution, this indicated the chemical formula C_16_H_20_O_5_ corresponding to an increase of a CH_2_ group compared to **1**–**3**. 1D and 2D NMR experiments of **4** revealed a clear resemblance to those of **2**–**3**, with the addition of a methoxy singlet (C^16^) with ^13^C and ^1^H resonances at 49.6 and 3.30 ppm, respectively (Table 1 and Figure 3). HMBC correlations from H^16^ to C^13^ (^3^*J*_CH_) and to C^12^ (^4^*J*_CH_) suggested that the C^13^ − OH of **2**–**3** was replaced with an O-methyl in **4**. This correlates with the loss of a methanol in the [M − CH_4_O + H]^+^ ion adduct and could possibly explain why **4** does not exert molecular rearrangement like **1**–**3**. The relative stereo configuration was assessed by ROESY correlations and observed to be identical to that of **3**.

### 2.3. Spiroleptosphol V (**5**)

**Figure 4:**
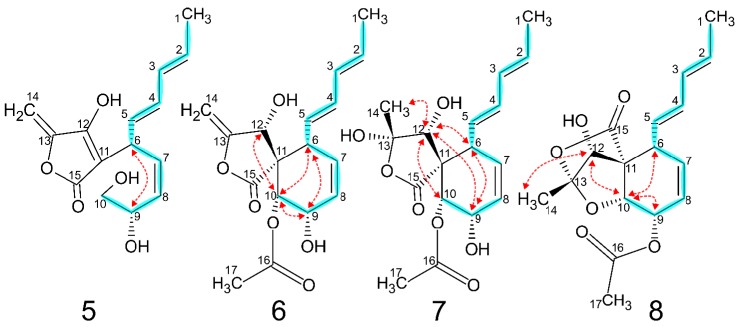
Diagnostic 2D NMR of **5**–**8** showing COSY (blue) and ROESY (red) correlations.

HR-ESI(+)MS of **5** identified a [M + H]^+^ at 279.1224 Da and a [M + Na]^+^ at 301.1040 Da. Additionally, two ion adducts from loss of water were observed at 261.1116 and 243.1011 Da. From this the chemical formula was deduced as C_15_H_18_O_5,_ identical to those of **1**–**3**. 1D and 2D NMR experiments of **5** showed some resemblance to C^1^-C^9^ of **1**–**4** based on chemical shifts and COSY correlations, however, a methylene C^10^ was observed in **5** which was a methine in **1**–**4** (Table 2 and Figure 4). ^1^H–^13^C HMBC connectivities from H^10^ were only observed to C^9^ and C^8^ indicating a primary alcohol at C^10^. C^6−8^ showed HMBC connectivity to a quaternary carbon C^11^ resonating at 103.9 ppm, and C^6^ showed additional ^3^*J*_CH_ HMBC correlations with two other quaternary carbons C^12^ and C^15^ resonating at 166.7 and 172.3 ppm, respectively. Three hydrogens had not been assigned, two of which were part of a CH_2_ group C^14^ with ^1^H chemical shifts resonating at 4.91 and 5.06 ppm and a ^13^C chemical shift resonating at 90.6 ppm suggesting a methylidene. Both H^14^ showed strong HMBC correlations to the quaternary C^13^ (δ_C_ 153.0 ppm) thus accounting for all carbon atoms. Three oxygens and one hydrogen needed to be assigned. Based on the previous information of chemical shifts, RDBE and HMBC correlations from H^14^ to C^11−13^ and C^15^ the structure of **5** was elucidated. A ROESY correlation observed between H^6^ and H^9^ suggests similar relative stereo configuration as described for **1**–**4**.

### 2.4. Spiroleptosphol Z (**6**)

HR-ESI(+)MS of **6** returned several ion adducts from which [M + H]^+^ was determined at 321.1324 Da based on a [M + Na]^+^ at 343.1146 Da resulting in the chemical formula C_17_H_20_O_6_. Additionally, three ion adducts were identified as [M − H_2_O + H]^+^, [M − CH_3_COOH + H]^+^ and [M − H_2_O − CH_3_COOH + H]^+^ at 303.1226, 261.1116 and 243.1007 Da, respectively, suggesting both an alcohol and an –OAc group being part of the molecule. 1D and 2D NMR experiments of **6** showed in part resemblance to those of **1**–**5** (Table 2 and Figure 4). COSY and HMBC correlations established C^1^-C^11^ to be similar to those of **1**–**4**. A methyl singlet resonating at 2.12 ppm and with HMBC correlations to C^16^ (δ_C_ 171.8 ppm) and C^9−10^ placed a –OAc group attached to C^10^ confirming the ion adducts observed from MS. HMBC and chemical shifts of C^12−15^ showed similarity to **5** except having a hydrogen at C^12^ in place of the double bond. This concluded the structure of **6**, where ROESY correlations revealed a similar relative stereo configuration to those previously described.

### 2.5. Spiroleptosphol Y (**7**)

HR-ESI(+)MS of **7** returned a [M + H]^+^ at 339.1443 Da and a [M + Na]^+^ ion adduct at 361.1256 Da. Ion adducts with neutral loss [M − H_2_O + H]^+^ and [M − H_2_O − CH_3_COOH + H]^+^ at 321.1335 and 261.1123 Da, respectively, were also observed. The mass spectrum was comparable to that of **6** with the addition of water, giving the chemical formula as C_17_H_22_O_7_. 1D and 2D NMR experiments of **7** showed similarity to those of **6** except around the C^13−14^ position (Table 2 and Figure 4). A singlet methyl (δ_H_ 1.6 ppm) with HMBC correlations to C^12−13^ suggested oxidation of the C^13^–C^14^ double bond giving the structure of **7**. ROESY cross peaks and ^1^H–^1^H coupling constants established the relative stereo configuration identical to that of **3** and **4**.

### 2.6. Spiroleptosphol Y (**8**)

HR-ESI(+)MS of **8** returned a [M + H]^+^ at 321.1324 Da supported by a [M + Na]^+^ at 343.1140 Da giving the chemical formula C_17_H_20_O_6_. An ion adduct for [M − CH_3_COOH + H]^+^ at 261.1113 Da indicated a loss of an –OAc group. 1D and 2D NMR experiments of **8** were comparable to those of **7** for C^1−11^ except the –OAc group was positioned at C^9^ (Table 2 and Figure 4). The remaining C^12−15^, H^12^ and H^14^ chemical shifts and splittings were comparable to **7**, however, differences observed including HMBC correlations could only be explained by an intra molecular rearrangement of **7** resulting in the structure of **8**. The relative stereo configuration of **8** was examined by ROESY cross peaks and determined to be identical to that of **7**.

### 2.7. Absolute Configuration

**Figure 5:**
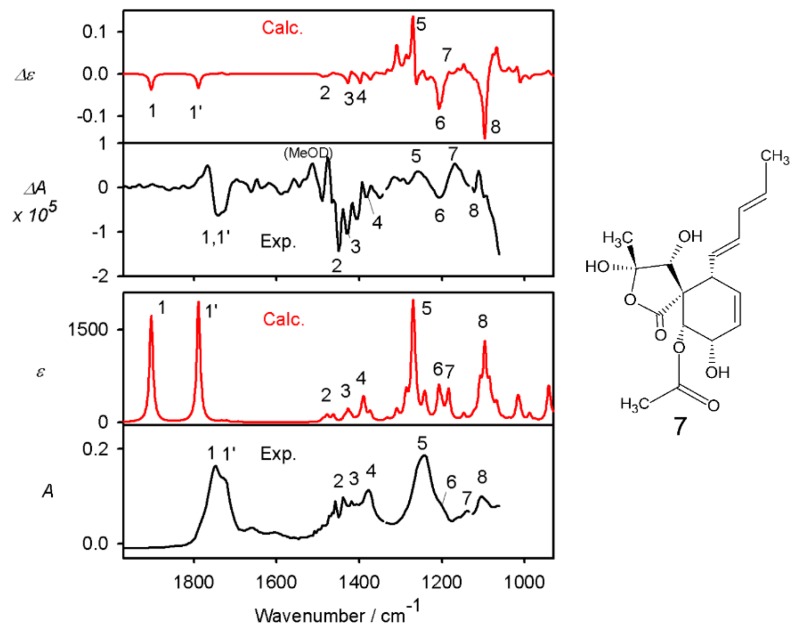
Calculated (red) and experimental (black) VCD (top) and IR (bottom) spectra of **7**, and the structure including annotation of the absolute configuration of **7** as used for the calculation. For easier orientation, corresponding bands are numbered. The large shift between the carbonyl vibrations (1 and 1’, calc. vs. exp.) is caused by the approximate solvent model in the calculations; the other two most intense IR bands (5 and 8) largely involve OH bending and CO stretching.

By NMR we thus could determine the relative configuration and some conformational parameters (e.g., the *E*-configuration of the double bonds in the conjugated chain). The absolute stereo configuration was established for **7** to be 6*R*,9*S*,10*R*,11*R*,12*R*,13*S* by vibrational circular dichroism (VCD), comparing the calculated spectrum to experimental curve (Figure 5).

As can be seen, the VCD experimental signal is rather noisy, due to the limited sample amount, and in some regions hampered by the absorption of the methanol (deuterated) solvent. Nevertheless, signs of all assignable VCD bands are consistently reproduced by the theory. The absolute configuration of the remaining compounds **1**–**6** and **8** are assumed to be identical to **7**, since the relative configuration for these are conserved it would not be likely that the fungi produces enantiomers where each chiral center would be inverted. Interestingly, the absolute configuration was identical to that described for spiroleptosphol from *L. doliolum* [12] for C^6^ and C^10−12^ but not for C^9^.

### 2.8. Biosynthetic Pathway

**Figure 6:**
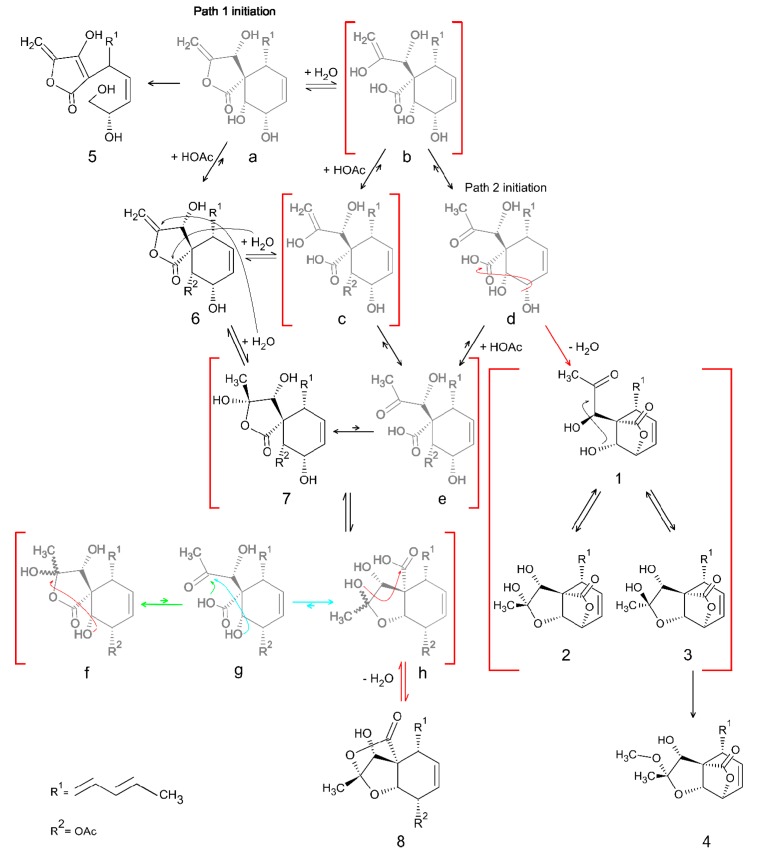
Proposed biosynthetic pathway for compounds **1**–**8**. All faded structures (**a**–**h**) are hypothetical intermediates which were not observed in this study. Red brackets indicate unstable intermediates or equilibria between compounds existing simultaneously. Equilibrium and reaction arrows are connected to the curved arrows of the respective color.

Based on the structures of **1**–**8** and the previously described biosynthetic pathway of spiroleptosphol from *L. doliolum* [24], a biosynthetic pathway of spiroleptosphols from *F. avenaceum* could be proposed with two paths for initiation (Figure 6).

Initiating path one with (a), hypothetical precursor to **6**, a rearrangement of the C^10^ –C^11^ bond into a double bond from C^11^–C^12^ would result in **5**. O-acetylation of (a) would produce **6**. Alternatively, the ester bond in (a) could be hydrolyzed leading to (b). O-acetylation of (b) would result in (c) which would be in equilibrium to **6**. Keto-enol tautomerism of the C^13^–C^14^ double bond in (b) yields a ketone at C^13^ and results in (d), which is the entry molecule for path two. Hydrolysis of the C^13^–C^14^ double bond of **6** leads to **7**. Opening the γ-lactone ring of **7** results in an equilibrium with (e), however, this reaction would most likely be pushed toward **7**. It is also possible to get (e) from rearrangement of (c) or from O-acetylation of (d). Relocation of the O-acetyl group of **7** and (e) from C^10^ to C^9^ would result in an equilibrium between (f) and (g), respectively. This equilibrium could also be pushed toward (h) through rearrangement of (g) to form an ether bond between C^10^ and C^13^. A condensation reaction of (h) would result in the formation of **8**, which is not possible for (f) because of sterical restraints. An alternative route could be proposed from (d), in which a condensation reaction would lead to the equilibrium of **1**–**3**. O-methylation of the C^13^ alcohol of **3** would explain the formation of **4**.

## 3. Materials and Methods

The genome sequenced *F. avenaceum* strain 05,001 isolated from Finnish grains was available from previous studies [25]. Fungal spores were prepared as previously described by Sørensen in 2013 and diluted with sterile filtered 15% glycerol in Milli-Q water to 1 × 10^6^ spores/mL and stored at –80 °C [3]. Compounds **1**–**8** were purified from three individual experimental setups.

Setup 1 (compounds **1**–**5**): A starter culture was made by inoculating 10 µL spores on a solid banana medium plate (200 g/L ecological yellow-brown banana including peel (*Musa acuminata,* Cavendish cultivar subgroup Grand Nain) was blended and added to 1 L Milli-Q water with 15 g/L agar) incubated at 25 °C for 14 days in the dark. 150 plates of solid banana medium were three point inoculated and incubated at 25 °C in the dark for 14 days. The agar plates were blended and secondary metabolites were extracted by covering the mycelia with ethyl acetate:dichloromethane:methanol with 1% formic acid and sonicated in a water bath for 40 minutes. The extract was filtered from the mycelia through MiraCloth (Calbiochem, Merck Millipore, Billerica, MA, USA) and the solvent was removed by rotary evaporation at 40 °C. The dried metabolites were re-dissolved in 3.5 mL methanol. The sample was centrifuged at 10,000× *g* for 5 minutes prior to HPLC-HRMS analysis.

The extract (10 µL injection volume) was analyzed by HPLC-DAD-HRMS using a Hitachi LaChrome Elite (Hitachi Ltd., Tokyo, Japan) HPLC system equipped with a pump (L-2130), autosampler (L-2200), column oven at 40 °C (L-2300) with a Hexyl-Phenyl column (150 × 4.6 mm Ascentis Xpress 2.7 μm, Sigma-Aldrich, St. Louis, MO, USA) and a DAD detector (L-2450) recording from 190 to 900 nm. The setup is coupled to a high-resolution mass spectrometer (Bruker compact MS ESI-qTOF, Bruker Daltonics, Bremen, Germany) (operated in positive mode with capillary: 4500 V, end plate offset 500 V, 4 L/min dry gas at 200 °C) through a 5:95 flow splitter. The gradient was run at 1 mL/min and initiated with 10% solvent A (LCMS -grade acetonitrile (HiPerSolv, VWR, Herlev, Denmark) with 0.1% formic acid (98%, Sigma-Aldrich)) and 90% solvent B (LCMS-grade water (HiPerSolv, VWR) with 0.1% formic acid) increasing linearly to 100% solvent A over 20 minutes and held for 10 minutes. Twenty µL sodium formate solution (10 mM NaOH and 26 mM formic acid in 1:1 MS-grade H_2_O:isopropanol) was injected 0.2 minutes after sample injection and used for mass spectrum calibration. The HPLC and mass spectrometer was controlled by HyStar v. 3.2 (Bruker Daltonics) and the data was analyzed in Compass DataAnalysis v. 4.2 (Bruker Daltonics).

Purification of compounds **1**–**5** was achieved by a two-step purification. Initially the metabolite extract was pre-fractionated using a semi-preparative 1260 Infinity HPLC system (Agilent Technologies) equipped with a DAD VL detector (Agilent Technologies, Santa Clara, CA, USA) and a Luna C18 LC column (5 µm, 250 × 10 mm, Phenomenex) kept at 40 °C. The gradient system increased from 10% solvent C (acetonitrile with 0.005% TFA) and 90% solvent D (Milli-Q water with 0.005% TFA) to 100% solvent C over 12 minutes, held at 100% for 2 minutes and returned to initial conditions over 2 minutes. The initial condition was run for 6 minutes before next injection. The flow was kept constant at 5 mL/minute and the injection volume was set to 100 µL. Thirteen fractions containing peaks observed at 234 nm eluting between 5.5 and 10.7 minutes were collected over 33 injections. Each of the collected fractions were frozen and lyophilized to dryness and re-dissolved in 200–400 µL LCMS-grade methanol. The samples were centrifuged at 14,100× *g* and transferred to 1.5 mL HPLC vials with 200 µL inserts. Two µL of each sample were analyzed as described previously by HPLC-DAD-HRMS using the same setting. Purification of the compounds was performed by manual collection from 30 µL injections by following the UV trace at 234 nm, where the waste-flow was redirected to 15 mL glass vials when peaks were observed to elute. Compounds **1**–**3** eluted as one peak and were collected in the same fraction. Compounds **4** and **5** both eluted as individual peaks. Collected fractions were frozen with liquid nitrogen and dried by lyophilization. The dried samples were re-dissolved in 550 µL deuterated methanol-d_4_ and transferred to 5 mm NMR tubes and analyzed by NMR. The ^1^H NMR spectra of (**1**–**8**) are in the Supplementary Materials.

Setup 2 (compound **6**): Five additional plates containing solid banana media were inoculated with 10 µL spores. These were incubated for 7 days at 25 °C in the dark. The agar plates were diced (approx. 0.3 × 0.3 cm), otherwise extraction proceeded as described previously. The dried sample was re-dissolved in 1.3 mL methanol and centrifuged at 14,100× *g* for 5 minutes. The supernatant was transferred to a HPLC vial and 12 runs of 100 µL were injected into the semi-preparative 1260 Infinity HPLC system with a flow of 5 mL/min. The gradient initiated at 20% solvent C and 80% solvent D, increasing to 100% solvent C over 30 minutes and returning to initial conditions over the next 3 minutes. Compound **6** was collected between 11.3 and 11.5 minutes. The collected fraction was lyophilized and re-dissolved in 40 µL deuterated methanol-d_4_ and transferred to a 1.7 mm NMR tube and analyzed by NMR.

Setup 3 (compounds **7**–**8**): Thirty plates of solid banana medium were inoculated with 10 µL spores and incubated at 25 °C in the dark for 14 days. The agar plates were diced (approx. 0.3 × 0.3 cm) and the mycelia were covered with ethyl acetate containing 1% formic acid. The following extraction proceeded as described for setup 2. The dried metabolites were re-dissolved in 4 mL methanol. The sample was centrifuged at 10,000× *g* for 5 minutes prior to HPLC analysis. The extract was analyzed by HPLC-DAD-HRMS using 10 µL injection volume. Compounds **7** and **8** were collected using the semi-preparative 1260 Infinity HPLC system applying two different gradients. Two mL of the sample were separated using a gradient initiating at 30% solvent C otherwise with the same settings described for setup 2. Compound **8** was collected between 7.2–7.5 minutes. The remaining 2 mL were separated using the same settings described for setup 2 and compounds **7** and **8** were collected between 7.65–7.95 and 11.4‒11.7 minutes, respectively. All collected fractions were frozen in liquid nitrogen and lyophilized. The two samples containing compound **8** were pooled into 1 mL methanol-d_4_ and transferred to a 5 mm NMR tube. Compound **7** was re-dissolved in 40 µL methanol-d_4_ and transferred to a 1.7 mm NMR tube. Both samples were analyzed by NMR.

Structure elucidation was achieved by NMR using a Bruker AVIII-600 MHz NMR spectrometer (Bruker BioSpin, Fällanden, Switzerland) equipped with a cryogenically cooled probe with z-gradients and all spectra were recorded in deuterated methanol at 298.1 K. All samples were analyzed with ^1^H-NMR (zg30), multiplicity-edited 2D-[^1^H-^13^C]-HSQC (pulse program: hsqcedetgpsisp2.3), double-quantum filtered 2D [^1^H-^1^H]-COSY (pulse program: cosygpmfph) and a non-decoupled 2D-[^1^H-^13^C]-HMBC (pulse program: hmbcetgpl3nd). A ROESY (pulse program: roesyetgp.2) experiment was recorded for compounds **4**–**8**. Compounds **1**–**3** were additionally analyzed with a NOESY (pulse program: noesygpphpr) and a TOCSY (pulse program: mlevetgppr) experiment. All spectra were recorded and analyzed using Topspin 3.5 (Bruker) and all ^13^C chemical shifts were evaluated by the CSEARCH-Robot-Referee [26].

For the vibrational spectra measurement, the sample of 1 mg of **7** in 50 µL of methanol was loaded into a BaF_2_ cell of 15 µm pathlength. VCD and IR spectra were measured with a Chiral IR-2X VCD spectrometer (BioTools, Inc., Jupiter, FL, USA) as blocks of 2048 scans at 8 cm^−1^ resolution. In total, 3 blocks were acquired and subsequently averaged to increase S/N ratio. Solvent spectra measured at identical conditions were subtracted as a baseline.

For the lowest-energy conformer of **7** the IR and VCD spectra were calculated at the B3PW91/6-31++G** approximation level with the COSMO (methanol) solvent model using the Gaussian program suite [27,28,29].

## 4. Conclusions

We have demonstrated that new polyketides can be induced by exotic growth media such as bananas, and that it is important to continue the search for original growth media in order to explore non-discovered secondary metabolite clusters from fungi. In summary, we isolated and elucidated the structure of eight analogous compounds spiroleptosphol T1, T2 and U―Z by mass spectrometry and NMR spectroscopy. The relative stereo configuration was established by NMR and the absolute configuration of spiroleptosphol Y was solved using vibrational circular dichroism. Finally, we proposed a biosynthetic pathway which includes all eight compounds described through our results. So far, no biological activity of the spiroleptosphols could be found.

The production of this whole cluster of compounds can easily be imagined to be the results of a single polyketide synthase leading to the initial product (a) or **6**. The chemistry leading to those two compounds is in agreement for the proposed biosynthetic pathways for spiroleptosphols from *L. doliolum* [24].

## Figures and Tables

**Table 1:** NMR spectroscopic data (600 MHz, methanol-d_4_, 298.1 K) of spiroleptosphol U (**1**), T1 (**2**), T2 (**3**) and W (**4**).

	**Spiroleptosphol U (1)**				**Spiroleptosphol T1 (2)**			
**#**	**δ_C_**	**type**	**δ_H_ (*J* in Hz)**	**HMBC ^2^**	**δ_C_**	**type**	**δ_H_ (*J* in Hz)**	**HMBC ^2^**
1	18.2	CH_3_	1.74 ^1^	2–4,6,7,11	18.2	CH_3_	1.74 ^1^	2–6,7,11
2	131.5	CH	5.71 ^1^	1,4–6	130.7	CH	5.70 ^1^	1,4,5
3	131.9	CH	6.03 ^1^	1,4–6	132.3	CH	6.06 ^1^	1,4,5
4	136.6	CH	6.05 ^1^	1–3,6	134.7	CH	6.10 ^1^	2,3,5,6,11
5	127.5	CH	5.24 (ddd, 1.3;9.6;14.4)	1–3,6,7,11	128.1	CH	5.51 (dd, 7.9;14.9)	2,3,6,7,11
6	46.5	CH	3.64 (ddd, 2.7;9.6)	4,7–12,15	47.9	CH	3.44 ^1^	4,5,7–12,15
7	135.4	CH	5.61 (ddd, 1.0;2.9;9.2)	5,6,9–12	136.1	CH	5.86 (dd, 2.6;9.4)	5,6,8–12
8	127.6	CH	6.16 (ddd, 2.7;5.8;9.2)	5–7,9–11	128.3	CH	6.25 (ddd, 2.6;5.9;9.4)	4–7,9–11
9	79.2	CH	4.52 (d, 5.8)	7,8,10–12,15	75.2	CH	4.75 (dd, 5.9)	6–8,10–13,15
10	78.6	CH	4.51 (s)	6,11–13,15	82.7	CH	4.41 (s)	6,8,9,11,12,14,15
11	59.0	C			59.0	C		
12	75.7	CH	4.36 (s)	6,10,11,13,15	86.0	CH	4.33 (s)	6,11,13–15
13	212.6	C			109.6	C		
14	28.4	CH_3_	2.35 (s)	12	24.0	CH_3_	1.34 (s)	6,11,12,13
15	176.0	C			177.9	C		
	**Spiroleptosphol T2 (3)**				**Spiroleptosphol W (4)**			
**#**	**δ_C_**	**type**	**δ_H_ (*J* in Hz)**	**HMBC ^2^**	**δ_C_**	**type**	**δ_H_ (*J* in Hz)**	**HMBC ^2^**
1	18.2	CH_3_	1.74 ^1^	2,4–6,11	18.2	CH_3_	1.74 (dd, 1.3;6.8)	2–5
2	130.3	CH	5.69 ^1^	1,4,5	130.7	CH	5.70 (dq, 6.7;14.6)	1,4,5
3	132.5	CH	6.06 ^1^	1,3,5,6,11	132.3	CH	6.05 (ddq, 1.5;10.5;14.7)	1,4,5
4	134.4	CH	6.09 ^1^	2,6	134.7	CH	6.11 (dd, 10.4;15.0)	2,3,5–7,11
5	128.5	CH	5.59 ^1^	3,6,7,11	128.1	CH	5.50 (dd, 7.9;14.9)	2,3,6,7,11
6	48.2	CH	3.43 ^1^	4,5,7–12,15	48.0	CH	3.44 (ddd, 2.5;2.5;8.0)	4,5,7,11,12,15
7	136.0	CH	5.82 (dd, 2.5;9.3)	5,6,8–12	136.2	CH	5.85 (ddd, 0.6;2.7;9.4)	5,6,9–12
8	128.8	CH	6.23 (ddd, 2.7;5.8;9.5)	4–7,9–11	128.2	CH	6.25	5–7,9–11
9	75.7	CH	4.73 (d, 5.8)	6–8,10–12,15	75.0	CH	4.79 (dd, 0.6;5.9)	6–8,10,11,15
10	83.5	CH	4.24 (s)	6,8,9,12–15	83.0	CH	4.31 (s)	6,8,9,11,12,15
11	57.1	C			58.9	C		
12	81.9	CH	4.17 (s)	6,11,13–15	86.0	CH	4.32 (s)	6,11,13–15
13	104.7	C			112.8	C		
14	25.9	CH_3_	1.48 (s)	11–13	18.9	CH_3_	1.30 (s)	12,13
15	177.2	C			177.8	C		
16					49.6	CH_3_	3.30 (s)	12,13

^1^ Partially overlapping or obscured ^1^H resonance; ^2 1^H-^13^C HMBC correlations with H→C directionality.

**Table 2:** NMR spectroscopic data (600 MHz, methanol-d4, 298.1 K) of spiroleptosphol V (**5**), Z (**6**), Y (**7**) and X (**8**).

	**Spiroleptosphol V (5)**				**Spiroleptosphol Z (6)**			
**#**	**δ_C_**	**type**	**δ_H_ (*J* in Hz)**	**HMBC ^2^**	**δ_C_**	**type**	**δ_H_ (*J* in Hz)**	**HMBC ^2^**
1	17.9	CH_3_	1.71 (d, 7.3)	2–5	18.0	CH_3_	1.73 (dd, 1.4;6.8)	2–6,11
2	128.8	CH	5.62 (dq, 6.9;14.2)	1,4–6	130.7	CH	5.66 (dq, 6.7;15.0)	1,3–5
3	132.4	CH	6.03 ^1^	1,4,5	132.0	CH	5.95 (ddq, 1.5;10.3;15.0)	1,4,5
4	131.5	CH	6.05 ^1^	2,3,6	135.5	CH	6.06 (dd, 10.3;15.2)	1–3,5,6,11
5	131.0	CH	5.71 (dd, 6.9;14.5)	1–3,6,11	128.6	CH	5.51 (dd, 8.7;15.2)	2–4,6,7,11
6	36.2	CH	4.30 (dd, 7.1;9.6)	5,7,8,11,12,15	40.1	CH	3.43 (ddd, 2.1:2.4;8.7)	4,5,8,10–12,15
7	132.5	CH	5.89 (ddd, 1.1;9.8;11.0)	4,6,9,10,11	130.3	CH	5.63 (ddd, 1.7;2.8;10.2)	5,6,9–11
8	129.9	CH	5.36 (ddd, 1.0;8.5;11.0)	5,6,9,10,11	128.3	CH	5.82 (ddd, 2.6;3.8;10.2)	3,5–7,9–11
9	69.4	CH	4.56 (dddd, 1.2;4.5,7.0,8.3)	7,8,10	64.3	CH	4.39 (ddd, 1.8;3.6;6.9)	6–8,10,11,15
10	66.9	CH_2_	3.40 (dd, 7.0;11.2)	8,9	72.0	CH	5.16 (d, 5.0)	6–9,11,12,15,16
			3.44 (dd, 4.5;11.2)	8,9				
11	103.9	C			55.2	C		
12	166.7	C			70.9	CH	4.94 (dd, 2.3;2.6)	6,10,11,13–15
13	153.0	C			158.9	C		
14	90.6	CH_2_	4.91 (d, 2.6)	6,11–13	89.2	CH_2_	4.76 (dd, 2.6;2.6)	10–13,15
			5.06 (d, 2.6)	6,12,13,15			4.63 (dd, 2.3;2.6)	10–13,15
15	172.3	C			174.6	C		
16					171.8	C		
17					20.7	CH_3_	2.12 (s)	9,10,16
	**Spiroleptosphol Y (7)**				**Spiroleptosphol X (8)**			
**#**	**δ_C_**	**type**	**δ_H_ (*J* in Hz)**	**HMBC ^2^**	**δ_C_**	**type**	**δ_H_ (*J* in Hz)**	**HMBC ^2^**
1	18.0	CH_3_	1.71 (d, 6.5)	2,3	17.9	CH_3_	1.74 (d, 6.7)	2–5
2	129.0	CH	5.60 ^1^	1	129.3	CH	5.67 (dq, 6.8;14.8)	1,3–5
3	132.7	CH	5.99 ^1^	1	132.4	CH	6.06 (dd, 14.8;10.5)	4,7
4	133.1	CH	6.00 ^1^	6	134.1	CH	6.21 (dd, 10.4;15.3)	2,3,6,7,11
5	131.9	CH	5.64 ^1^	1,3	128.5	CH	5.96 (dd, 8.5;15.3)	2–4,6,7,11
6	40.3	CH	3.65 ^1^	4,5	41.6	CH	3.32 ^1^	
7	131.5	CH	5.59 ^1^	6,9,11	137.4	CH	5.91 (d, 10.0)	5,6,8,9,11
8	127.6	CH	5.58 ^1^	6,9,11	122.8	CH	5.74 ^1^	5–7,9–11
9	65.5	CH	4.39 ^1^		63.9	CH	5.42 (dd, 4.6;4.6)	7,8,10,11,16
10	73.3	CH	5.31 (d, 3.8)	6,8,9,11,15,16	77.0	CH	4.38 (d, 4.3)	6,9,11–13,15,17
11	52.5	C			56.5	C		
12	77.5	CH	4.01 (s)	6,10,11,14	83.8	CH	4.23 (s)	6,10,13,15
13	104.7	C			111.9	C		
14	25.0	CH_3_	1.60 (s)	12,13	14.8	CH_3_	1.55 (s)	6,10–13,15
15	175.9	C			174.6	C		
16	172.6	C			171.7	C		
17	21.2	CH_3_	2.10 (s)	16	20.4	CH_3_	2.01 (s)	9,16

^1^ Partially overlapping or obscured ^1^H resonance; ^2 1^H-^13^C HMBC correlations with H→C directionality.

## Supplementary Materials

The following are available online, Figure S1: 1D ^1^H-NMR spectra of (**1**–**5**), Figure S2: 1D ^1^H-NMR spectra of (**6**–**8**).
