# Supplementary material for: Characterization of Eight Novel Spiroleptosphols from Fusarium avenaceum
Source: Molecules. 2019 Sep 26;24(19):3498. doi: 10.3390/molecules24193498 (PMC6804164; doi:10.3390/molecules24193498)
Supplement: Supplementary file 1 [file molecules-24-03498-s001.pdf]

Supplementary Materials: Characterization of eight novel spiroleptosphols from *Fusarium avenaceum*

Klaus Ringsborg Westphal, Manuela Ilse Helga Werner, Katrine Amalie Hamborg Nielsen, Jens Laurids Sørensen, Valery Andrushchenko, Jacob Winde, Morten Hertz, Mikkel Astrup Jensen, Mathilde Lauge Mortensen, Petr Bouř, Teis Esben Sondergaard and Reinhard Wimmer

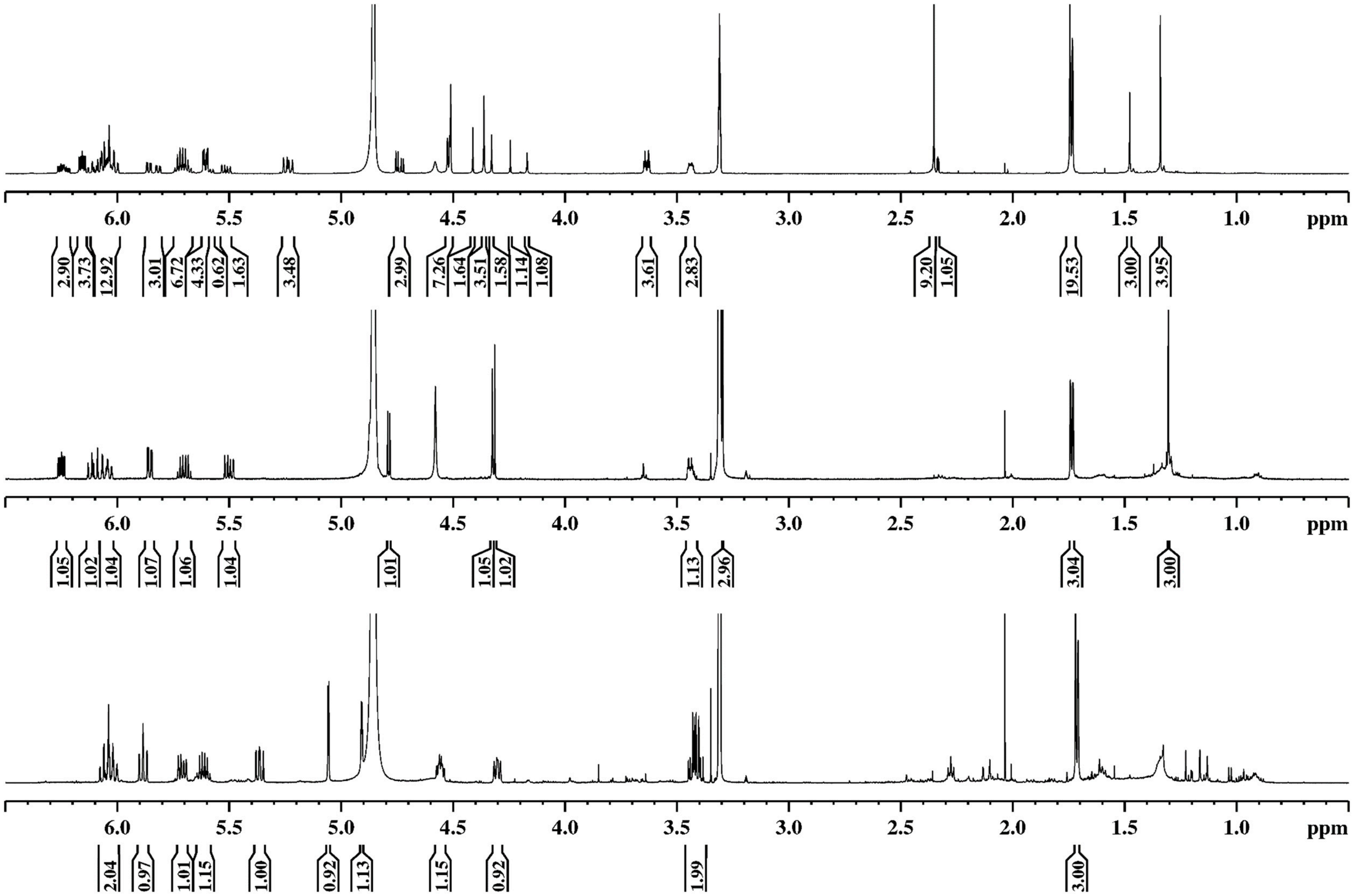

Supplementary Figure S1. 1D ^{1}H-NMR spectra of (1-5). Spectra were recorded on a 600 MHz spectrometer in deuterated methanol-d4 and the ppm axis was calibrated according to the solvent chemical shift at 3.31ppm. Integrals were calibrated by setting a methyl signal to three hydrogens. a, ^{1}H spectrum of the equilibrium mixture of spiroleptosphol U, T1 and T2 (**1-3**). b, ^{1}H spectrum of spiroleptosphol W (**4**). c, ^{1}H spectrum of spiroleptosphol V (**5**).

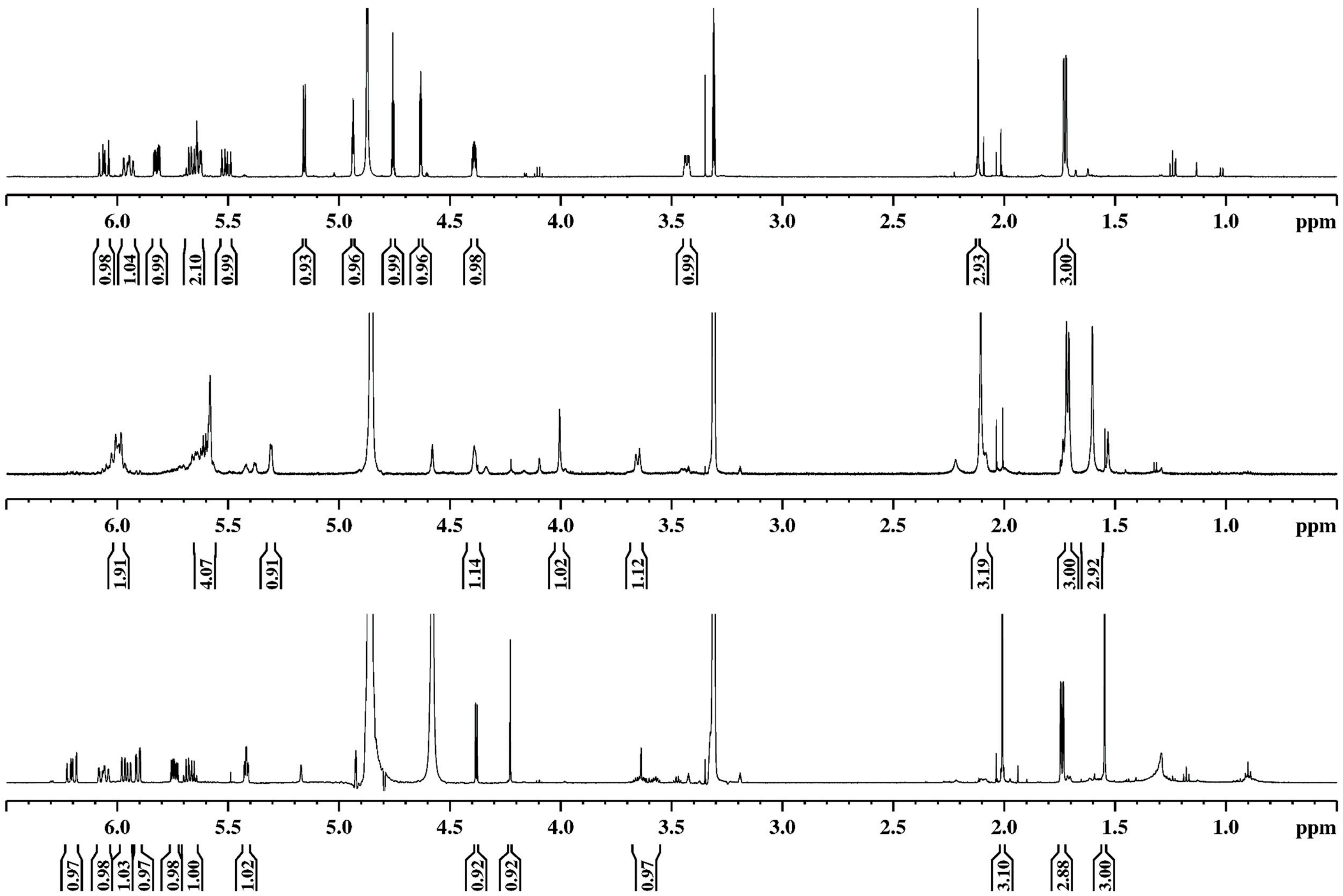

Supplementary Figure S2. 1D ¹H-NMR spectra of (6-8). Spectra were recorded on a 600 MHz spectrometer in deuterated methanol-d4 and the ppm axis was calibrated according to the solvent chemical shift at 3.31ppm. Integrals were calibrated by setting a methyl signal to three hydrogens. **b**, ^{1}H spectrum of spiroleptosphol Z (**6**). **b**, ^{1}H spectrum of spiroleptosphol Y (**7**). **c**, ^{1}H spectrum of spiroleptosphol X (**8**).
